# Are Men’s Perceptions of Sexually Dimorphic Vocal Characteristics Related to Their Testosterone Levels?

**DOI:** 10.1371/journal.pone.0166855

**Published:** 2016-11-22

**Authors:** Michal Kandrik, Amanda C. Hahn, Joanna Wincenciak, Claire I. Fisher, Katarzyna Pisanski, David R. Feinberg, Lisa M. DeBruine, Benedict C. Jones

**Affiliations:** 1 Institute of Neuroscience and Psychology, University of Glasgow, Glasgow, United Kingdom; 2 School of Psychology, University of Sussex, Brighton, United Kingdom; 3 Department of Psychology, Neuroscience, & Behaviour, McMaster University, Hamilton, ON, Canada; Universidad de Chile, CHILE

## Abstract

Feminine physical characteristics in women are positively correlated with markers of their mate quality. Previous research on men’s judgments of women’s facial attractiveness suggests that men show stronger preferences for feminine characteristics in women’s faces when their own testosterone levels are relatively high. Such results could reflect stronger preferences for high quality mates when mating motivation is strong and/or following success in male-male competition. Given these findings, the current study investigated whether a similar effect of testosterone occurs for men’s preferences for feminine characteristics in women’s voices. Men’s preferences for feminized versus masculinized versions of women’s and men’s voices were assessed in five weekly test sessions and saliva samples were collected in each test session. Analyses showed no relationship between men’s voice preferences and their testosterone levels. Men’s tendency to perceive masculinized men’s and women’s voices as more dominant was also unrelated to their testosterone levels. Together, the results of the current study suggest that testosterone-linked changes in responses to sexually dimorphic characteristics previously reported for men's perceptions of faces do not occur for men's perceptions of voices.

## Introduction

Feminine physical characteristics are positively correlated with measures of women’s reproductive health (e.g., [1,2]), general medical health [3, 4], and maternal tendencies [5]. Given that these traits are highly valued in mates, women displaying feminine physical characteristics tend to be judged as more attractive than relatively masculine women (reviewed in [6, 7]).

Several lines of evidence suggest that men’s preferences for feminine characteristics in women’s faces are stronger when their own testosterone levels are relatively high. For example, Welling et al. [8] reported that men showed stronger preferences for feminine shape characteristics in women’s, but not men’s, faces when their own testosterone levels were higher. Relatedly, Welling et al. [9] found that men who had been randomly allocated to the winning condition in a male-male contest (playing against another man in a video game with a fixed outcome) subsequently showed stronger preferences for feminine shape characteristics in women’s faces than did men randomly allocated to the losing condition. Welling et al. [9] did not measure men’s testosterone levels. However, given that testosterone levels tend to be higher in winners of male-male contests than in losers (reviewed in [10]), Welling et al’s [9] results are consistent with men showing stronger preferences for feminine women when their own testosterone levels are relatively high.

Increased preferences for feminine women when men’s own testosterone levels are high could occur because success in male-male competition increases access to high quality mates [9]. Given that testosterone levels are associated with mating motivation in men (see [11] for a recent review), increased preferences for feminine women when men’s own testosterone levels are high could also reflect stronger preferences for high quality mates when men’s mating motivation is strong [8].

To date, evidence that men show stronger preferences for feminine women when their own testosterone levels are high has come exclusively from studies investigating men’s preferences for feminine characteristics in women’s faces. However, sexually dimorphic characteristics are also present in the human voice (reviewed in [6, 7]). Women’s voices tend to have both higher fundamental frequencies (i.e., higher pitch) and higher formant frequencies than do men’s voices (reviewed in [6, 7]). These feminine acoustic characteristics are associated with attractiveness in women’s voices [7, 12–14] and men tend to respond to femininity in women’s faces and voices in similar ways (reviewed in [7]). Because previous research suggests that men’s preferences for femininity in women’s faces are stronger when their own testosterone levels are high [8, 9], the current study used a longitudinal design to investigate whether men’s preferences for higher voice pitch and higher formant frequencies in women’s voices are stronger when their own salivary testosterone levels are high. Additionally, because previous research has reported that men show stronger preferences for feminine characteristics in women’s, but not men’s, faces when their own testosterone levels are high [8], we also assessed men’s preferences for manipulated pitch and formant frequencies in men’s voices. Men’s voice preferences were tested in five weekly test sessions, with each participant also providing a saliva sample in each test session.

While men tend to ascribe high attractiveness to women’s voices with feminine acoustic properties (reviewed in [7]), men tend to ascribe high dominance to men’s and women’s voices with masculine characteristics (e.g., low pitch and formants, reviewed in [15]). Moreover, previous research has shown that voices contain cues to men’s and women’s physical dominance [16, 17]. Research on men’s dominance judgments of men’s faces suggests that winners of male-male contests are less likely to ascribe high dominance to masculine men than are losers [18]. Welling et al. [19] recently proposed that this effect of contest outcome on men’s perceptions of other men’s dominance could be due to the effects of testosterone on men’s dominance perceptions. Consequently, the current study also tested whether men were more likely to ascribe high dominance to men’s voices with masculine characteristics when their own testosterone levels were relatively low. We also examined men’s dominance judgments of women’s voices.

## Methods

### Participants

Forty-six heterosexual men participated in the study (mean age = 22.1 years, SD = 3.20 years). All participants were students at the University of Glasgow (Scotland, UK). None of these men were currently taking any form of hormonal supplement and all indicated that they had not taken any form of hormonal supplement in the 90 days prior to participation. One additional man was tested but excluded from the dataset because of an average hormone level that was more than five standard deviations above the sample mean. All participants provided written consent and all aspects of the study were approved by the School of Psychology (University of Glasgow) ethics committee.

### Voice stimuli

Recordings of 6 men and 6 women between the ages of 18 and 25 speaking the English monopthong vowels, “ah”/ɑ/, “ee”/i/, “e”/ɛ/, “oh”/o/, and “oo”/u/, were made in an anechoic sound-controlled booth using a Sennheiser MKH 800 cardioid condenser microphone, at an approximate distance of 5–10 cm. Voice recordings were digitally encoded using an M-Audio Fast Track Ultra interface at a sampling rate of 96 kHz and 32-bit amplitude quantization, and transferred to a computer as PCM WAV files using Adobe Soundbooth CS5 version 3.0.

Following other recent work on perceptions of sexually dimorphic vocal characteristics (e.g., [20]), we created two feminized and two masculinized versions of each original voice recording by independently manipulating voice pitch or formants using the Pitch-Synchronous Overlap Add (PSOLA) algorithm in Praat version 5.2.15 [21]. Pitch was raised (feminized) or lowered (masculinized) by 10% from baseline while holding formants constant. Likewise formants were raised (feminized) or lowered (masculinized) by 10% from baseline while holding pitch constant. This process created 12 pairs of voices (6 male and 6 female) that differed in pitch and 12 pairs of voices that differed in formants (6 male and 6 female). Following these manipulations, we amplitude normalized the sound pressure level of all voices to 70 decibels using the root mean squared method. The male voice stimuli used in the current study have previously been used to investigate hormonal correlates of women’s preferences for masculine characteristics in men’s voices [20]. Voice pitch and formant measures for the feminized and masculinized voice stimuli are given in our supplemental materials (Table A and Table B in S1 Supplementary Materials).

### Procedure

All participants completed five weekly test sessions which took place between 2pm and 5pm to minimize diurnal variation in hormone levels [22]. During each test session, participants provided a saliva sample via the passive drool method [22]. Participants were instructed to avoid consuming alcohol and coffee in the 12 hours prior to participation and to avoid eating, smoking, drinking, chewing gum, or brushing their teeth in the 60 minutes prior to participation. Saliva samples were immediately frozen and stored at -32°C until being shipped, on dry ice, to the Salimetrics Lab (Suffolk, UK) for analysis, where they were assayed using the Salivary Testosterone Enzyme Immunoassay Kit 1–2402 (M = 177.69 pg/mL, SD = 40.22 pg/mL). Although previous research examining links between men’s hormone levels and responses to sexually dimorphic characteristics has focused on possible effects of testosterone levels [8, 9, 19], research on mating motivation [23, 24] and male-male competition [25, 26] more generally has also implicated cortisol. Consequently, men’s saliva samples were also assayed using the Salivary Cortisol Enzyme Immunoassay Kit 1–3002 (M = 0.19 μg/dL, SD = 0.08 μg/dL). All assays passed Salimetrics’ quality control.

In each of five test sessions, participants listened to 24 pairs of voices (each pair consisting of a masculinized and a feminized version of the same voice) through headphones and, on separate trials, reported which voice in each pair sounded either more attractive or more dominant. Male and female voice stimuli were presented in separate blocks of trials and attractiveness and dominance judgments were also made in separate blocks of trials. Block order, trial order, and the order in which participants listened to the masculinized and feminized versions in each pair were fully randomized. The sound volume was set to the same level on every testing machine, and participants were asked not to change it. All testing machines as well as all headphones used in the study were identical. This type of test has been used to assess perceptions of masculinized versus feminized versions of voices in previous work (e.g., [13, 20]). Data are included as S1 Data.

## Results

First, we calculated the proportion of trials on which feminized versions of women’s voices or masculinized versions of men’s voices were chosen. This score was calculated separately for each combination of participant, test session, judgment (attractiveness, dominance), manipulation type (pitch manipulation, formant manipulation), and sex of voice (male, female). These scores were centered on 0.5 (i.e., chance).

Next, we investigated how these scores were related to men’s current hormone levels. Attractiveness judgments of women’s voices, attractiveness judgments of men’s voices, dominance judgments of women’s voices, and dominance judgments of men’s voices were all analyzed separately.

In each analysis, we tested for effects of hormone levels on voice perceptions using multilevel modeling with test sessions grouped by participant (five test sessions per participant). Analyses were conducted using R [27], *lme4* [28], and *lmerTest* [29]. For analyses of responses to women’s voices, the proportion of feminized voices chosen (centered on chance) was entered as the dependent variable at the test session level. For analyses of responses to men’s voices, the proportion of masculinized voices chosen (centered on chance) was entered as the dependent variable, again at the test session level. Testosterone and cortisol levels were entered as predictors at the test session level, each centered on their subject-specific means. Manipulation type (effect-coded so that the pitch manipulation was assigned a value 0.5 and the formant manipulation was assigned a value -0.5) was also entered as a predictor at the test session level. Each model also included two-way interactions between current testosterone level and manipulation type and between current cortisol level and manipulation type. The analyses and results are specified in full in Results in S1 Supplementary Materials.

### Attractiveness judgments of women’s voices

In our analysis of women’s vocal attractiveness, the intercept approached significance (t = 1.86, p = .070), indicating that men generally preferred feminized versions of women’s voices to masculinized versions. There were no other significant effects or interactions (all |t| < 1.10, all p >.274). Repeating this analysis with testosterone retained as a predictor, but excluding cortisol, or with cortisol retained as a predictor, but excluding testosterone, did not reveal any effects involving hormone levels (all |t| < 0.970, all p > .333).

### Attractiveness judgments of men’s voices

In our analysis of men’s vocal attractiveness, the intercept was significant (t = 7.01, p < .001), indicating that men generally preferred masculinized versions of men’s voices to feminized versions. The effect of manipulation type was also significant (t = 5.40, p < .001), indicating that men showed stronger preferences for masculinized male voices manipulated in pitch (M = 0.18, SD = 0.23) than manipulated in formants (M = 0.09, SD = 0.23). There were no other significant effects or interactions (all |t| < 1.40, all p > .161). Repeating this analysis with testosterone retained as a predictor, but excluding cortisol, or with cortisol retained as a predictor, but excluding testosterone, did not reveal any effects involving hormone levels (all |t| < 1.69, all p > .093).

### Dominance judgments of women’s voices

In our analysis of women’s vocal dominance, the intercept was significant (t = –9.73, p < .001), indicating that men generally judged masculinized versions of women’s voices to be more dominant than feminized versions. The effect of manipulation type was also significant (t = – 4.23, p < .001), indicating that men chose masculinized female voices as the more dominant more often when voices were manipulated in pitch (M = – 0.24, SD = 0.22) than when they were manipulated in formants (M = – 0.16, SD = 0.26). There were no other significant effects or interactions (all |t| < 0.52, all p > .610). Repeating this analysis with testosterone retained as a predictor, but excluding cortisol, or with cortisol retained as a predictor, but excluding testosterone, did not reveal any effects involving hormone levels (all |t| < 0.54, all p > .590).

### Dominance judgments of men’s voices

In our analysis of men’s vocal dominance, the intercept was significant (t = 14.36, p < .001), indicating that men generally judged masculinized versions of men’s voices to be more dominant than feminized versions. There were no other significant effects or interactions (all |t| < 1.25, all p > .241). Repeating this analysis with testosterone retained as a predictor, but excluding cortisol, or with cortisol retained as a predictor, but excluding testosterone, did not reveal any effects involving hormone levels (all |t| < 1.06, all p > .290).

## Discussion

The current study tested for possible relationships between within-subject changes in men’s salivary testosterone and cortisol levels and their preferences for, and dominance perceptions of, voices manipulated in sexually dimorphic acoustic properties. Consistent with previous research, men generally judged masculinized male and female voices as more dominant than feminized versions [15] and judged masculinized male voices as more attractive than feminized versions [13]. Also consistent with previous research [12, 30], men tended to judge feminized female voices as more attractive than masculinized versions, although this effect of femininity only approached significance in the current study (p = .070). The weak preference for feminized versions of women’s voices in the current study is likely a consequence of our manipulation of acoustic characteristics of voices (20% difference between feminized and masculinized versions) being very similar to the just-noticeable difference for men’s judgments of women’s vocal attractiveness (18% difference) reported by Re et al. [31]. This was done to avoid men’s preferences for feminized versions of women’s voices being at ceiling and masking potential relationships with hormone levels.

In contrast to our findings, a recent study found that within-subject changes in estradiol predicted women’s preferences for vocal masculinity in men’s voices [20]. This apparent sex difference in hormonal modulation of voice preferences may potentially reflect overall differences in mating strategies, as women may use more and finer-grained information about potential mates, or may be more sensitive to cues of quality, in order to offset potentially greater costs to their fitness associated with poor partner choice [32]. The extent to which hormone-linked changes in social judgments of voices could be driven by effects of steroidal hormones other than testosterone on hearing is not known.

Previous research has suggested that men’s preferences for feminine characteristics in women’s, but not men’s, faces become stronger when their testosterone levels are high [8, 9]. By contrast with these results for men’s face preferences, the current study observed no significant effect of testosterone on men’s preferences for sexually dimorphic characteristics in either women’s or men’s voices. Previous research has also suggested that the tendency to ascribe dominance to men displaying masculine facial characteristics might also be greater when men’s own testosterone levels are low [18, 19]. However, the current study observed no significant effect of testosterone on men’s dominance perceptions of either women’s or men’s voices. We also observed no effects of cortisol on men’s responses to sexually dimorphic vocal characteristics when judging the attractiveness or dominance of voices. Although previous research suggested that social perceptions of sexually dimorphic characteristics in voices are very similar to those reported in the face perception literature [30, 33], it is possible that using more socially relevant stimuli (e.g., sentences) could produce effects of hormones on voice perception that were not apparent in the current study. The results of the current study suggest that hormone-linked changes in responses to sexually dimorphic characteristics that have previously been reported for men's perceptions of faces [8, 9] do not occur for men's perceptions of voices.

## Supporting Information

S1 Analysis Script (R)

S1 Data (CSV)

S1 Supplementary Materials (PDF)
