# Supplementary material for: Are Men’s Perceptions of Sexually Dimorphic Vocal Characteristics Related to Their Testosterone Levels?
Source: PLoS One. 2016 Nov 22;11(11):e0166855. doi: 10.1371/journal.pone.0166855 (PMC5119782; doi:10.1371/journal.pone.0166855)
Supplement: S1 Supplementary Materials — (PDF) [file pone.0166855.s003.pdf]

**S1 Supplementary Materials**

**Are men's perceptions of sexually dimorphic vocal characteristics related to their testosterone levels? (Kandrik et al.)**

**Table A** in S1 Supplementary Materials Mean (SEM) of voice pitch and formant measures from feminized and masculinized male voice stimuli (given in Hz).

**Table B** in S1 Supplementary Materials. Mean (SEM) of voice pitch and formant measures from feminized and masculinized female voice stimuli (given in Hz).

**Results** in S1 Supplementary Materials

**Table A** in S1 Supplementary Materials

| Manipulation | *F*0 | *F*1 | *F*2 | *F*3 | *F*4 | *F*n |
|---|---|---|---|---|---|---|
| Masculinized Pitch | 111<br>(5) | 457<br>(12) | 1525<br>(44) | 2567<br>(57) | 3440<br>(104) | 1997<br>(49) |
| Feminized Pitch | 135<br>(6) | 460<br>(11) | 1525<br>(42) | 2571<br>(58) | 3437<br>(104) | 1998<br>(48) |
| Masculinized Formants | 123<br>(5) | 421<br>(8) | 1375<br>(43) | 2351<br>(57) | 3145<br>(103) | 1823<br>(48) |
| Feminized Formants | 123<br>(5) | 513<br>(11) | 1682<br>(47) | 2817<br>(59) | 3756<br>(109) | 2192<br>(49) |

**Acronyms:** *F*0 = fundamental frequency (pitch); *F*1-*F*4 = first to fourth formant; *F*n = mean formant frequency (an average of *F*1-*F*4). Mean *F*0 was measured using Praat's autocorrelation algorithm with a search range set to 65-300 Hz. Formants *F*1-*F*4 were measured using the Burg Linear Predictive Coding algorithm. Formants were first overlaid on a spectrogram and manually adjusted until the best visual fit of predicted onto observed formants was obtained. All acoustic measurements were taken from the central, steady-state portion of each vowel, averaged across vowels for each voice, and then averaged across voices. This was done separately for each type of masculinity manipulation.

**Table B in S1 Supplementary Materials**

| Manipulation | *F*0 | *F*1 | *F*2 | *F*3 | *F*4 | *F*n |
|---|---|---|---|---|---|---|
| Masculinized Pitch | 194<br>(6) | 862<br>(31) | 2029<br>(35) | 3154<br>(47) | 4203<br>(35) | 2562<br>(27) |
| Feminized Pitch | 237<br>(8) | 867<br>(39) | 2035<br>(33) | 3160<br>(48) | 4214<br>(32) | 2596<br>(28) |
| Masculinized Formants | 216<br>(7) | 849<br>(39) | 1999<br>(47) | 3083<br>(58) | 4123<br>(41) | 2513<br>(41) |
| Feminized Formants | 216<br>(7) | 892<br>(31) | 2027<br>(46) | 3168<br>(41) | 4220<br>(35) | 2577<br>(28) |

**Acronyms:** *F*0 = fundamental frequency (pitch); *F*1-*F*4 = first to fourth formant; *F*n = mean formant frequency (an average of *F*1-*F*4). Mean *F*0 was measured using Praat's autocorrelation algorithm with a search range set to 100-600 Hz. Formants *F*1-*F*4 were measured using the Burg Linear Predictive Coding algorithm. Formants were first overlaid on a spectrogram and manually adjusted until the best visual fit of predicted onto observed formants was obtained. All acoustic measurements were taken from the central, steady-state portion of each vowel, averaged across vowels for each voice, and then averaged across voices. This was done separately for each type of masculinity manipulation.

## Results in S1 Supplementary material
## Variable legend

```
fc_c = The forced-choice preference score (proportion of masculine male or feminine female voices chosen, centered on chance=0.5)
test.c = subject-mean centered testosterone (pg/mL)
cort.c = subject-mean centered cortisol (µg/mL)
manip.e = voice manipulation (effect-coded so pitch = +0.5, formant = -0.5)
```

## Full model predicting preferences for women's voices

```
Formula: fc_c ~ test.c * manip.e + cort.c * manip.e + (1 | id_code/session)

     AIC      BIC   logLik deviance df.resid
   -50.2    -13.0     34.1    -68.2      451

Scaled residuals:
     Min       1Q   Median       3Q      Max
-2.35557 -0.63426  0.05982  0.67281  2.15551

Random effects:
 Groups          Name        Variance  Std.Dev.
 session:id_code (Intercept) 2.592e-18 1.610e-09
 id_code         (Intercept) 1.347e-02 1.161e-01
 Residual                    4.387e-02 2.095e-01
Number of obs: 460, groups:  session:id_code, 230; id_code, 46

Fixed effects:
                Estimate   Std. Error        df  t value  Pr(>|t|)
(Intercept)     3.659e-02  1.970e-02  4.600e+01   1.857   0.0697 .
test.c          2.653e-04  4.166e-04  4.140e+02   0.637   0.5247
manip.e        -6.518e-03  1.953e-02  4.140e+02  -0.334   0.7388
cort.c          1.036e-01  1.329e-01  4.140e+02   0.780   0.4359
test.c:manip.e  9.134e-04  8.332e-04  4.140e+02   1.096   0.2736
manip.e:cort.c -2.790e-01  2.657e-01  4.140e+02  -1.050   0.2944
------
```

## Model predicting preferences for women's voices (testosterone only)

```
Formula: fc_c ~ test.c * manip.e + (1 | id_code/session)

     AIC      BIC   logLik deviance df.resid
   -52.5    -23.6     33.2    -66.5      453

Scaled residuals:
     Min       1Q   Median       3Q      Max
-2.38096 -0.61498  0.06103  0.67577  2.20396

Random effects:
 Groups          Name        Variance  Std.Dev.
 session:id_code (Intercept) 2.608e-18 1.615e-09
 id_code         (Intercept) 1.345e-02 1.160e-01
 Residual                    4.405e-02 2.099e-01
Number of obs: 460, groups:  session:id_code, 230; id_code, 46

Fixed effects:
                Estimate  Std. Error         df  t value  Pr(>|t|)
(Intercept)     3.659e-02  1.970e-02  4.600e+01   1.857   0.0697 .
test.c          3.466e-04  4.042e-04  4.140e+02   0.858   0.3916
manip.e        -6.521e-03  1.957e-02  4.140e+02  -0.333   0.7392
test.c:manip.e  6.944e-04  8.083e-04  4.140e+02   0.859   0.3908
------
```

**Model predicting preferences for women's voices (cortisol only)**

```
Formula: fc_c ~ cort.c * manip.e + (1 | id_code/session)

     AIC      BIC   logLik deviance df.resid
   -52.6    -23.7     33.3    -66.6      453

Scaled residuals:
     Min       1Q   Median       3Q      Max
-2.33887 -0.65613  0.07894  0.65009  2.17349

Random effects:
 Groups          Name        Variance Std.Dev.
 session:id_code (Intercept) 0.00000  0.0000
 id_code         (Intercept) 0.01345  0.1160
 Residual                    0.04404  0.2099
Number of obs: 460, groups:  session:id_code, 230; id_code, 46

Fixed effects:
                 Estimate   Std. Error       df  t value   Pr(>|t|)
(Intercept)      0.036593   0.019703  46.000000   1.857   0.0697 .
cort.c           0.124806   0.128882 414.000000   0.968   0.3334
manip.e         -0.006519   0.019570 414.000000  -0.333   0.7392
cort.c:manip.e  -0.206042   0.257765 414.000000  -0.799   0.4246
---
```

## Full model predicting preferences for men's voices

```
Formula: fc_c ~ test.c * manip.e + cort.c * manip.e + (1 | id_code/session)

     AIC      BIC   logLik deviance df.resid 
  -108.3    -71.1     63.1   -126.3      451 

Scaled residuals: 
     Min       1Q   Median       3Q      Max 
-2.46190 -0.67824  0.01972  0.67716  2.50080 

Random effects:
 Groups          Name        Variance  Std.Dev. 
 session:id_code (Intercept) 3.203e-16 1.790e-08
 id_code         (Intercept) 1.316e-02 1.147e-01
 Residual                    3.834e-02 1.958e-01
Number of obs: 460, groups:  session:id_code, 230; id_code, 46

Fixed effects:
                Estimate   Std. Error        df  t value  Pr(>|t|)    
(Intercept)     1.348e-01  1.922e-02  4.600e+01   7.013 8.71e-09 ***
test.c          5.465e-04  3.894e-04  4.140e+02   1.403    0.161    
manip.e         9.855e-02  1.826e-02  4.140e+02   5.397 1.14e-07 ***
cort.c          1.142e-01  1.242e-01  4.140e+02   0.920    0.358    
test.c:manip.e  6.826e-04  7.789e-04  4.140e+02   0.876    0.381    
manip.e:cort.c -8.094e-02  2.484e-01  4.140e+02  -0.326    0.745    
---
```

## Model predicting preferences for men's voices (testosterone only)

```
Formula: fc_c ~ test.c * manip.e + (1 | id_code/session)

     AIC      BIC   logLik deviance df.resid 
  -111.3    -82.4     62.7   -125.3      453 

Scaled residuals: 
     Min       1Q   Median       3Q      Max 
-2.47535 -0.68325  0.01075  0.66603  2.48639 

Random effects:
 Groups          Name        Variance  Std.Dev. 
 session:id_code (Intercept) 2.724e-16 1.651e-08
 id_code         (Intercept) 1.315e-02 1.147e-01
 Residual                    3.843e-02 1.960e-01
Number of obs: 460, groups:  session:id_code, 230; id_code, 46

Fixed effects:
               Estimate   Std. Error       df  t value  Pr(>|t|)    
(Intercept)    1.348e-01  1.922e-02 4.600e+01   7.013 8.71e-09 ***
test.c         6.362e-04  3.775e-04 4.140e+02   1.685   0.0927 .  
manip.e        9.855e-02  1.828e-02 4.140e+02   5.391 1.18e-07 ***
test.c:manip.e 6.191e-04  7.549e-04 4.140e+02   0.820   0.4126    
---
```

**Model predicting preferences for men's voices (cortisol only)**

```
Formula: fc_c ~ cort.c * manip.e + (1 | id_code/session)

     AIC      BIC   logLik deviance df.resid 
  -109.6    -80.6     61.8   -123.6      453 

Scaled residuals: 
     Min       1Q   Median       3Q      Max 
-2.46184 -0.66800  0.03588  0.65166  2.50976 

Random effects:
 Groups          Name        Variance  Std.Dev.
 session:id_code (Intercept) 1.518e-16 1.232e-08
 id_code         (Intercept) 1.313e-02 1.146e-01
 Residual                    3.859e-02 1.965e-01
Number of obs: 460, groups:  session:id_code, 230; id_code, 46

Fixed effects:
                Estimate   Std. Error      df  t value  Pr(>|t|)    
(Intercept)      0.13478    0.01922  46.00000   7.013 8.71e-09 ***
cort.c           0.15787    0.12064 414.00000   1.309    0.191    
manip.e          0.09855    0.01832 414.00000   5.380 1.25e-07 ***
cort.c:manip.e  -0.02643    0.24129 414.00000  -0.110    0.913    
---
```

**Full model predicting dominance perceptions of women's voices**

```
Formula: fc_c ~ test.c * manip.e + cort.c * manip.e + (1 | id_code/session)
   Data: data.dom.f

     AIC      BIC   logLik deviance df.resid
   -53.1    -15.9     35.5    -71.1      451

Scaled residuals:
    Min      1Q  Median      3Q     Max
-2.5408 -0.6616 -0.1513  0.5771  2.8569

Random effects:
 Groups          Name        Variance  Std.Dev.
 session:id_code (Intercept) 1.667e-17 4.082e-09
 id_code         (Intercept) 1.506e-02 1.227e-01
 Residual                    4.317e-02 2.078e-01
Number of obs: 460, groups:  session:id_code, 230; id_code, 46

Fixed effects:
               Estimate   Std. Error         df  t value  Pr(>|t|)
(Intercept)    -1.996e-01  2.052e-02  4.600e+01  -9.727 9.75e-13 ***
test.c          2.007e-04  4.133e-04  4.140e+02   0.486    0.628
manip.e        -8.188e-02  1.938e-02  4.140e+02  -4.226 2.93e-05 ***
cort.c         -4.000e-02  1.318e-01  4.140e+02  -0.304    0.762
test.c:manip.e  4.266e-04  8.265e-04  4.140e+02   0.516    0.606
manip.e:cort.c  5.974e-03  2.636e-01  4.140e+02   0.023    0.982
---
```

## Model predicting dominance perceptions of women’s voices (testosterone only)

```
Formula: fc_c ~ test.c * manip.e + (1 | id_code/session)

     AIC      BIC   logLik deviance df.resid
   -57.0    -28.1     35.5    -71.0      453

Scaled residuals:
    Min      1Q  Median      3Q     Max
-2.5289 -0.6596 -0.1509  0.5761  2.8565

Random effects:
 Groups          Name        Variance Std.Dev.
 session:id_code (Intercept) 0.00000  0.0000
 id_code         (Intercept) 0.01506  0.1227
 Residual                    0.04318  0.2078
Number of obs: 460, groups:  session:id_code, 230; id_code, 46

Fixed effects:
               Estimate    Std. Error        df  t value  Pr(>|t|)
(Intercept)    -1.996e-01  2.052e-02  4.600e+01  -9.727 9.75e-13 ***
test.c          1.693e-04  4.001e-04  4.140e+02   0.423    0.672
manip.e        -8.188e-02  1.938e-02  4.140e+02  -4.226 2.93e-05 ***
test.c:manip.e  4.313e-04  8.003e-04  4.140e+02   0.539    0.590
---
```

## Model predicting dominance perceptions of women's voices (cortisol only)

```
Formula: fc_c ~ cort.c * manip.e + (1 | id_code/session)

     AIC      BIC   logLik deviance df.resid 
   -56.6    -27.7     35.3    -70.6      453 

Scaled residuals: 
    Min      1Q  Median      3Q     Max 
-2.5558 -0.6564 -0.1418  0.5883  2.8560 

Random effects:
 Groups          Name        Variance Std.Dev.
 session:id_code (Intercept) 0.00000  0.0000  
 id_code         (Intercept) 0.01505  0.1227  
 Residual                    0.04322  0.2079  
Number of obs: 460, groups:  session:id_code, 230; id_code, 46

Fixed effects:
                Estimate   Std. Error      df  t value  Pr(>|t|)    
(Intercept)     -0.19963    0.02052  46.00000  -9.727 9.75e-13 ***
cort.c          -0.02398    0.12768 414.00000  -0.188    0.851    
manip.e         -0.08188    0.01939 414.00000  -4.223 2.96e-05 ***
cort.c:manip.e   0.04003    0.25536 414.00000   0.157    0.876    
---
```

## Full model predicting dominance perceptions of men’s voices

```
Formula: fc_c ~ test.c * manip.e + cort.c * manip.e + (1 | id_code/session)

     AIC      BIC   logLik deviance df.resid 
  -157.8   -120.6     87.9   -175.8      451 

Scaled residuals: 
    Min      1Q  Median      3Q     Max 
-3.4863 -0.5036  0.1761  0.6273  2.2254 

Random effects:
 Groups          Name        Variance Std.Dev.
 session:id_code (Intercept) 0.004358 0.06601 
 id_code         (Intercept) 0.011438 0.10695 
 Residual                    0.030787 0.17546 
Number of obs: 460, groups:  session:id_code, 230; id_code, 46

Fixed effects:
                 Estimate Std. Error         df  t value  Pr(>|t|)    
(Intercept)     2.627e-01  1.829e-02  4.600e+01  14.362   <2e-16 ***
test.c         -1.718e-04  3.953e-04  1.840e+02  -0.435    0.664    
manip.e         1.087e-02  1.636e-02  2.300e+02   0.664    0.507    
cort.c          3.882e-02  1.261e-01  1.840e+02   0.308    0.758    
test.c:manip.e  6.040e-04  6.980e-04  2.300e+02   0.865    0.388    
manip.e:cort.c -2.773e-01  2.226e-01  2.300e+02  -1.246    0.214    
---
```

## Model predicting dominance perceptions of men's voices (testosterone only)

```
Formula: fc_c ~ test.c * manip.e + (1 | id_code/session)

     AIC      BIC   logLik deviance df.resid 
  -160.2   -131.2     87.1   -174.2      453 

Scaled residuals: 
    Min      1Q  Median      3Q     Max 
-3.4335 -0.5064  0.1547  0.6281  2.1625 

Random effects:
 Groups          Name        Variance Std.Dev.
 session:id_code (Intercept) 0.004264 0.0653  
 id_code         (Intercept) 0.011436 0.1069  
 Residual                    0.030994 0.1761  
Number of obs: 460, groups:  session:id_code, 230; id_code, 46

Fixed effects:
                 Estimate Std. Error         df  t value  Pr(>|t|)    
(Intercept)     2.627e-01  1.829e-02  4.600e+01  14.362   <2e-16 ***
test.c         -1.413e-04  3.828e-04  1.840e+02  -0.369    0.712    
manip.e         1.087e-02  1.642e-02  2.300e+02   0.662    0.509    
test.c:manip.e  3.863e-04  6.780e-04  2.300e+02   0.570    0.569    
---
```

## Model predicting dominance perceptions of men's voices (cortisol only)

```
Formula: fc_c ~ cort.c * manip.e + (1 | id_code/session)

     AIC      BIC   logLik deviance df.resid
  -160.9   -131.9     87.4   -174.9      453

Scaled residuals:
    Min      1Q  Median      3Q     Max
-3.4627 -0.5105  0.1774  0.6216  2.1988

Random effects:
 Groups          Name        Variance Std.Dev.
 session:id_code (Intercept) 0.004328 0.06579
 id_code         (Intercept) 0.011434 0.10693
 Residual                    0.030887 0.17575
Number of obs: 460, groups:  session:id_code, 230; id_code, 46

Fixed effects:
                Estimate Std. Error        df  t value  Pr(>|t|)
(Intercept)      0.26268    0.01829  46.00000  14.362   <2e-16 ***
cort.c           0.02511    0.12212 184.00000   0.206    0.837
manip.e          0.01087    0.01639 230.00000   0.663    0.508
cort.c:manip.e  -0.22909    0.21586 230.00000  -1.061    0.290
---
```
